# Social media in the learning ecologies of communications students: Identifying profiles from students’ perspective

**DOI:** 10.1007/s10639-022-11169-3

**Published:** 2022-06-24

**Authors:** Carles Bruguera, Montse Guitert, Teresa Romeu

**Affiliations:** Universitat Oberta de Catalunya (UOC), Rambla del Poblenou, 156, 08018 Barcelona, Spain

**Keywords:** Learning ecologies, Distance education, Online learning, Social media, Student profiling

## Abstract

Social media can be a support during the initial training of communication professionals, although most studies on social media and learning have mainly focused on other professional groups. The purpose of this article is to explore how communication students learn and their use of social media platforms, in order to identify the role of social media in supporting communication students’ learning. Data was collected using a questionnaire sent to communication students of the UOC and analyzed using a clustering technique, to identify student profiles based on how they organize their learning and their use of social media platforms. Our results suggest that there are 5 main student profiles: (i) students that learn through many contexts with strong support of Wikipedia, Blogs and YouTube; (ii) students with preference for academically guided learning resources; (iii) students with preference for informal and digital learning contexts, supported by social networks; (iv) students with preference for physical and formal contexts with a slight support of interactive social media platforms and (v) students detached academically with low use of learning resources and occasional use of social media platforms. Findings show that in the formative stage, there is a different degree of utility of social media among communication students, with a division between platforms that we could designate as more static and sources of information (Wikipedia, blogs or YouTube) and more interactive and dynamic (Twitter, Facebook or LinkedIn). The findings of this article can help to inform and make communication studies more flexible, collaborative and personalized oriented. In follow up studies, it would be interesting to delve further into how COVID-19 has affected the role of social media platforms.

## Introduction

Communication studies is an interdisciplinary field that integrates different professional disciplines such as journalism, audiovisual communication, advertising, or consulting, which focus their activity on three aspects: the production, dissemination, and consumption of information through diverse formats, media, and platforms (Marauri-Castillo et al., 2018). Professionals of this sector face several challenges to address the changes and innovations arising from digitalization (Hamad et al., 2017. As a result of the widespread use of digital technologies and social media, most citizens are producers, distributors, and consumers at the same time (We Are Social, 2020). As a result, communications professionals have experienced profound disruption in their professional trajectories as certain technologies have directly caused the obsolescence of numerous professional profiles (Salaverría, 2016). This has led to the emergence of new practices and professional profiles and a subsequent continuous effort of keeping up to date professionally, which is already a key aspect in the formative stage of communication professionals (Wobalis, 2018). These profiles have integrated in their professional activity tasks related to social media management, online marketing, or multimedia edition (Ewing et al., 2019). In addition to mastering technological tools, professionals must be clear about what they are for and how to use them professionally to respond to the instantaneousness, greater coverage, and massive communication (Prestridge, 2019).

The emergence of new multipurpose professional profiles oriented to digital communication directly affects the training of communication professionals (Casero-Ripollés et al., 2013, p.54). In this context “establishing a direct connection between university training and the expectations of the professional sector is unavoidable” (p. 55). The digital environment "calls for a rethinking of communication studies" (Barrios, 2014, p.168) where "both academia and professionals assume a process of constant updating and culturalization at a technological level; a process that not only implies mastering devices, but also learning to face new ways of thinking and acting” (p.173). In this scenario "it is necessary to highlight an important gap between the training received by advertising and communication professionals and the needs of the labor market" (Núñez et al., 2013, p.186).

Among digital technologies, social media platforms are an interactive, digital, collaborative, and instant form of communication that can support professionals to acquire knowledge, stay up-to-date and encourage collaboration and networking, connecting professionals who would not otherwise be linked (Barrot, 2020). Its proliferation brings undeniable benefits, such as access to a higher number of sources, dissemination of information content on a global scale or interaction with the audience (Malik et al., 2019). In addition, the fact that an important part of users has access to information at any time and place democratizes access to knowledge (Middleton & Beckingham, 2015). In contrast, these digital platforms contain a series of hazardous qualities such as false information, trolls or addiction that can hinder their use for learning and professional development purposes (Future Today Institute, 2018). Literature reviews on social media and learning show a recent, varied and growing interest in the field of research, as shown by systematic literature reviews such as those by Barrot (2020) or Bruguera et al. (2019), even though there are two professional sectors that dominate much of the scientific literature: the health and education sectors. In this sense, although there is a considerable amount of research that focuses on social media opportunities focused on the health (Curran et al., 2017; Sterling et al., 2017) and education (Manca & Ranieri, 2016; Tess, 2013) sector, there is little research that focuses on the university training stage of communication professionals.

The main objective of this study is to identify the role of social media in supporting communication students’ learning, in order to inform and improve the initial or continuing training offered by educational institutions. Two research questions arise from this objective:

- Research question 1: What profiles of students make up communication studies according to the way in which they organize their learning and their use of social media platforms?
- Research question 2: What role do social media platforms have in the training of communication students?

For this purpose, the learning ecologies concept constitutes an integrative framework of how students organize their learning (Jackson, 2019; Sangrà et al., 2019). A learning ecologies conceptual framework includes the set of contexts formed by configurations of activities, materials and relations that offer opportunities for learning (Peters & Romero, 2019; Romeu-Fontanillas et al., 2020). This set of components take place across different contexts within the formal-informal continuum and the physical-digital continuum. An ecological vision of learning arises from the “need to consider the rich, dynamic, interconnected, and complex systems in which knowledge is created and shared in today’s digital society” (González-Sanmamed et al., 2018, p.26). The learning ecology frame is also sensitive to the fact that digital technologies have expanded the learning opportunities available to professionals (Esposito et al., 2015), helping us to identify the role of social media in the learning process of communication students at the Open University of Catalonia (UOC) in the context of Spain. This identification and its consequences will allow us to make communication students aware of the components that make up their own learning ecology and at the same time encourage them to participate in new learning scenarios through social media.

We have decided to group all degrees of the UOC’s communication studies because, although career opportunities in the communication sector differ relatively in their profiles (advertising, media, consulting), all these communicators in training have gone from analog to digital, with a great impact of social media platforms in their professional practice. Profiling communication students based on how they organize their learning and their use of social media platforms has the ultimate purpose of informing educational institutions that offer different kinds of training (initial or continuing, from degrees to master’s degrees) for communication professionals. Therefore, our study provides parameters to improve the practice of these professionals and their professional updating challenges.

## Material and methods

### Survey

**Fig. 1:**
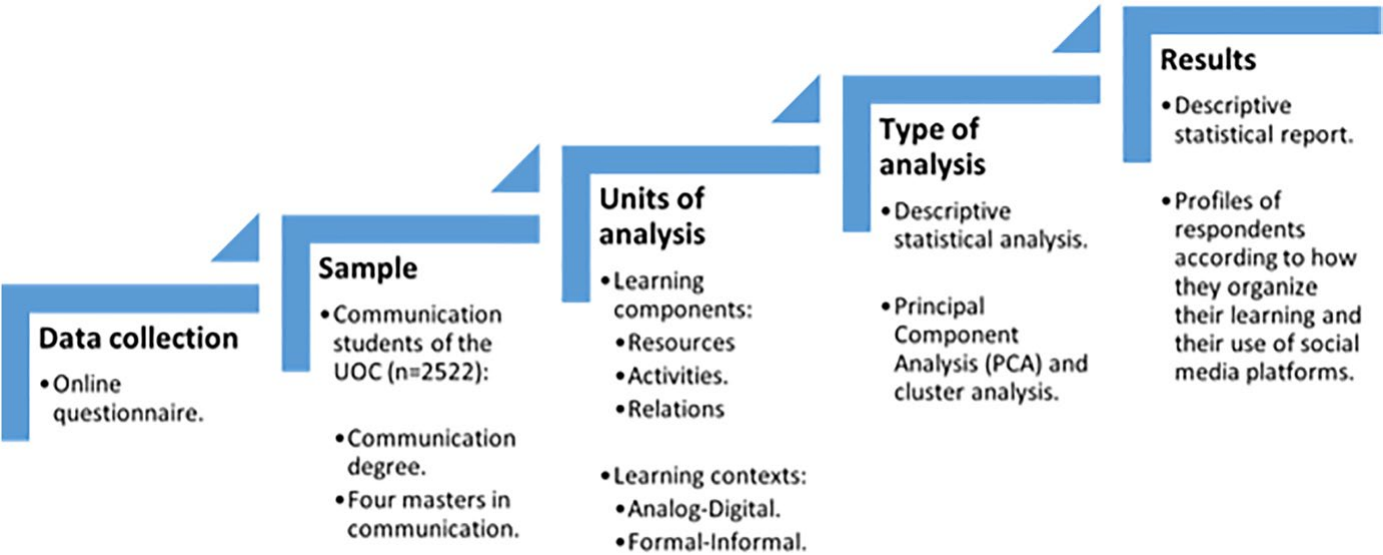
Data analysis plan

This study followed a survey design. Such a method is suitable for social research interested in collecting original data to describe a population too large to be directly observed (Babbie, 2013, p.2012). Our study used a non-experimental online questionnaire. As a research instrument, the questionnaire “produces quantitative data for its statistical treatment and analysis, asking questions in a structured way to a certain set of people, representing a certain population” (Fàbregues et al., 2016, p.27). The online questionnaire was used as a way to deepen our understanding of the role that social media plays in the learning ecologies of a large group of UOC communication students (Fig. 1).

### Sampling

An intentional sampling was used to select the sample of the questionnaire. This is a type of sampling in which the units to be observed are selected based on the researcher’s judgment on which will be most useful or representative (Babbie, 2013, p.187). The population included active UOC communication students during the second half of 2019. The purpose of using this type of sampling was to access a wide amount of distant learners in training in the communication field. An advantage of intentional sampling was to have access to a wide range of communication students through the UOC’s Learning Center area of this university. This was extremely time and cost-effective. In contrast, intentional sampling has the difficulty in having a significant representativeness of the larger population of interest. The final sample was made up of students of the following degrees:

- Communication degree.
- Master’s Degree in ‘Journalism and Digital Communication’.
- Master’s Degree in ‘Social Media: Management and Strategy’.
- Master’s Degree in ‘Strategy and Creativity in Advertising’.
- Master’s Degree in ‘Corporate Communication, Protocol and Events’.

Based on the data provided by the eLearn Center of the UOC university the total population of students was 2522, the final sample, or respondents, were 564 students and the confidence level was 95 (margin of error: 3,64) or 99 (margin of error: 4,79).

### Survey validation

Once the objectives of the survey were determined and the sample was selected, a process of constructing survey items began (Fàbregues et al., 2016, p.47). This construction was supported by a range of literature, which was helpful to provide the information needed for the formulation and proper wording of the survey items:

- A literature review on professional development in the communication field (Bruguera et al., 2019), the learning ecologies framework (Jackson, 2019; Sangrà et al., 2019; Romeu-Fontanillas et al., 2020), and learning and social media (Dron & Anderson, 2014; Middleton & Beckingham, 2015).
- Questionnaires used in other studies on learning and social media (Alsobayel, 2016; Hunter & Hall, 2017).
- Other surveys and questionnaires of projects that have addressed the concept of learning ecologies such as: Eco4Learn (Lifelong Learning Ecologies: ICT Contributions to Teacher Professional Development) and Eco4Learn-HE (How the best university teachers learn in the digital age: Impact of learning ecologies on the quality of teaching).

To validate and ensure the reliability of the instruments, we carried out an expert judgment method (Flick, 2007), a useful validation technique that is defined as "an informed opinion of people with a background on the subject, who are recognized by others as qualified experts in the topic, and who can give information, evidence, judgments and assessments" (Escobar-Pérez & Cuervo-Martínez, 2008). The validation process consisted of rating the criteria in terms of univocality, relevance and importance of the different items of the qüestionnaire. The questionnaire was revised with adaptations based on suggestions from various experts at UOC university. The experts have included members of the UOC research community, especially researchers from the Edul@b research group, professors of the UOC communication studies and PhD students in the program in Education and ICT of the same institution. Fourteen experts reviewed and made comments on the questionnaire by rating the criteria in terms of univocality, relevance and importance. The validation process can be seen in the final thesis dissertation (Bruguera, 2021). In addition, the Cronbach alpha served as an internal consistency coefficient and reliability analysis in order to calculate the degree of confidence towards our instrument. With a α of Cronbach of 0.926 in its ordinal items, the overall instrument showed excellent reliability.

### Data collection

The survey was administered by sending the questionnaire to the sample of students enrolled in the 5 distinct masters degrees. The database through the UOC’s eLearn Center was used because they have access to the email accounts of the 2,552 enrolled students. The administration of the survey was confidential, anonymous, and untraceable. The software used to design, send, and monitor the number of questionnaire responses was Qualtrics. Regarding the submission of the questionnaire, the eLearn Center laboratory sent a first email to students inviting them to participate in the study, anonymously, and attaching a link to the questionnaire. This email was sent at the end of May 2019. A month later, the center sent a second reminder email to students, which aimed to encourage participation among those students who had not yet answered the questionnaire. Finally, the possibility of participating in the questionnaire was open until July 2019, when the data analysis began.

### Statistical analysis

Descriptive statistics were used as a set of procedures that help organize, summarize, graphically represent, and describe quantitative data (Fàbregues et al., 2016; Babbie, 2013). As an extension of the descriptive statistical analysis, we conducted a Principal Component Analysis (PCA) to explore whether there is an underlying structure in the set of variables presented in the questionnaire and identify whether the different interval items in the questionnaire could be grouped into main components, as a way to reduce a dataset to a more manageable size while retaining as much original information as possible (Field, 2013 p.628, quoted in Peters, 2020). Next, the PCA allows for a cluster analysis to be performed. Cluster analysis aims to "provide groupings of sets of elements, objects, or behaviors that are similar to each other. The result of a cluster analysis provides the set of associations that exist across multiple groups that the analysis provides’’ (Allen, 2017, p.143). The cluster analysis (Garson, 2014, cited in Peters & Romero, 2019, p.157) helped us identify and profile students based on the components of their learning ecologies and their use of social media. Such a process is a useful method for transforming numerical data into correspondent profiles based on a narrative story.

## Results

### Principal component analysis

The PCA was carried out using SPSS. Varimax was used as a type of extraction for facilitating the interpretability of the factors because "orthogonal rotation methods assume that the factors in the analysis are not correlated" (Gorsuch, 1983, pp. 203–204, cited in Dean, 2009 p. 21). One solution that emerged from the PCA became the basis of the cluster analysis. The first step of the PCA was to check whether factor analysis could be applied to the data set obtained and to see the suitability of the 80 non-nominal items, on which we can potentially apply a factorial analysis, using the Kaiser-Meyer Olkin (KMO) and Bartlett tests. High values in KMO (about 1.0) generally indicate that a factorial analysis can be useful with the data, as well as the value close to 0.000 in the Bartlett sphericity test. Test results confirm that a factorial analysis was useful with our variables, reflecting a score of 0.894 which is considered remarkable in KMO with a value close to 0.000 of Bartlett’s sphericity test sufficient.

The PCA extracted 19 core component solutions spread over 4 large blocks that we show in Table 1. Eight of the main components made up the basis of the cluster analysis that we describe below for being closely related to the research questions.

**Table 1:** Overall PCA results

Survey block	Component Solution	Main components
Block 1: Use of social media	CS1. Frequency of social media use	C1: Social media with more static and organizational core content features (Wikipedia, Blogs, YouTube) C2: Social media with more social network features and interaction between users (Instagram, Facebook, LinkedIn, Twitter) C3: Pinterest
Block 2: Components for the upgrade	CS2. Frequency of actions for updating	C4: Mostly digital and slightly more informal actions C5: Face-to-face and formal actions
	CS3. Frequency of resources for updating	C6: Less formally guided digital resources C7: Mixed resources (analog/digital) more formally guided
	CS4. Frequency of interactions for updating	C8: Interactions in a non-academic context C9: Interactions in an academic context
Block 3: Utility of social media for updating	CS 5. Utility of social media for updating	C10: Social media utility of static and organizational core type of content (Wikipedia, Blogs, YouTube, Pinterest) C11: Usefulness of social network platforms and more personal orientation (Instagram, Facebook) C12: Usefulness of social network platforms and more professional guidance (LinkedIn, Twitter)
	CS 6. Usefulness of actions in social media for the update	C13: Usefulness of organization actions, monitoring and interaction with other users C14: Usefulness of search, query and subscription actions of contents C15: Usefulness of actions of elaboration and sharing of contents
	CS 7. Social Media Relationship Utility for Updating	C16: Utility of connections of the non-professional field C17: Usefulness of connections of the professional field
	CS 8. Social Media Opportunities for Updating	C18: Social media opportunities linked to the possibility of connecting with other users and the development and sharing of content C19: Social media opportunities linked to features related to flexibility, personalization, dynamism and open content

### Cluster analysis

The second step towards profiling respondents consisted of a cluster analysis to identify students based on a conglomeration (segmentation) technique. According to Allen (2017), "the choice of input variables becomes important both to provide the basis of similarity within a grouping and to differentiate the differences between groupings" (p.143).The final solution main components that have emerged from the PCA have been used as a basis for creating students profiles, using a clustering technique (segmentation), depending on the frequency of activities, resources and interactions they use for keeping up-to-date and their use of social media.

Finally, eight component variables were identified as suitable for responding to the purpose of the investigation, shown in Table 1. The first PCA block represents two components that group the use of distinctive social media platforms. Below, the PCA solutions that have been used in our process of clustering are presented, together with the associated questionnaire variables.

The next step of the cluster analysis consisted in establishing the total number of clusters of respondents. For this purpose, the methodology of previous studies that have used similar analytical procedures through SPSS was followed (Kahan et al., 2017; Guitert et al., 2018; Poellhuber et al., 2019). We have performed a hierarchical cluster analysis to define the possible cluster range. Once the analysis of hierarchical clusters was performed, we observed by means of a dendrogram as the number of clusters was mainly adjusted to 4, 5 and 6 possible or appropriate solutions. Secondly, we have followed the K-means procedure to form and set the definitive number of clusters. To determine the optimal solution, the analytical procedure tested 4, 5 and 6 categories, comparing the quality of the different models and the meanings of the profiles produced. A classification model was sought in which the profiles would be qualitatively and significantly different, while trying to preserve the quality of the final solution. Finally, the optimal number of clusters was set to 5. Table 2 shows the comparative characterization of the profiles of respondents identified through the cluster analysis based on the components that have emerged from the PCA (Table 3).

**Table 2:** PCA variables used for cluster analysis

Block	PCA solution	Variable Description
Using social media	C1: Social media with more static core and content organization features (Wikipedia, Blogs, YouTube)	> Frequency of Wikipedia use > Frequency of use of Blogs > Frequency of use of Youtube
	C2: Social media with more social networking features and user interaction (Instagram, Facebook, LinkedIn, Twitter)	> Frequency of Instagram use > Frequency of Facebook use > Frequency of LinkedIn use > Frequency of Twitter use
Learning actions and activities	C3: Mostly digital and slightly more informal stocks	> Informal conversations in virtual spaces (social networks, forums, chats) > Informal face-to-face conversations with others > Online courses > Online participation in events
	C4: Face-to-face and formal actions	> In-person courses > In-person participation in events
Learning resources	C5: Less formally guided digital resources	> Information found on the network (e.g., through Google-like search engines) > Social media posts > Specialized online resources
	C6: More formally guided mixed (analog/digital) resources	> Specialized resources in paper format > UOC Material
Learning relationships	C7: Interactions within a non-academic context	> Professional references > Digital Influencers > Workmates > Friends and family
	C8: Interactions within an academic context	> Teachers > Study colleagues

**Table 3:** Clusters and scores of the principal components

Cluster	n	%	C1	C2	C3	C4	C5	C6	C7	C8
1	108	19,1	0,99	0,16	0,75	0,87	0,59	0,44	0,86	0,57
2	107	19	0,06	-0,92	-0,07	-0,62	0,00	0,61	-0,91	0,59
3	113	20	-0–08	0,56	0,54	-0,65	0,73	-0,37	0,37	0,01
4	133	23,6	-0,52	0,33	-0,42	0,64	-0,55	0,33	0,14	-0,24
5	103	18,3	-0,34	-026	-0,76	-0,37	-0,71	-1,11	-0,55	-0,93

**Fig. 2:**
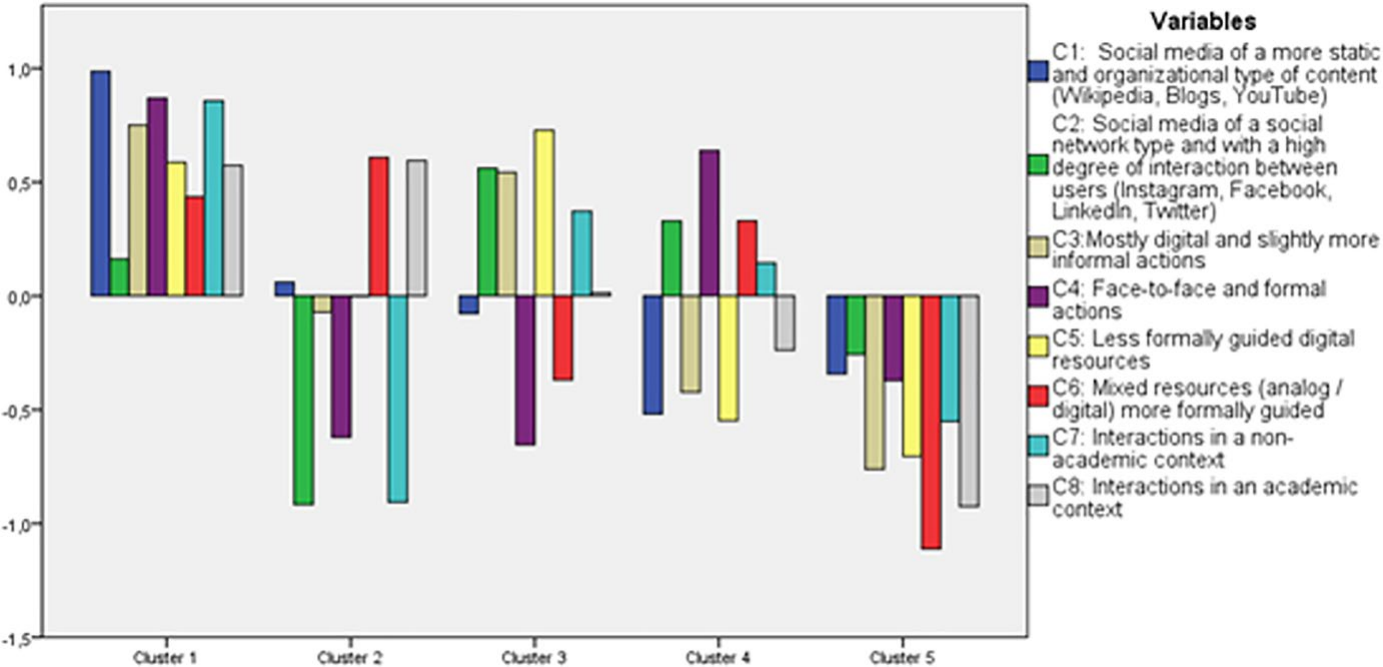
Visual representation of respondent clusters and average component scores

Figure 2 illustrates the different scores of the 5 different students’ profiles that have emerged from the *K-means* method solutions. The figure shows the profiles of respondents based on the resources, activities, and relations they use to learn and their use of social media platforms.

Cluster solutions have been named through an interpretive process and then compared to each other to maximize differences and similarities. Below we present a description of the learning profile solutions in light of the component variables.

Learner profile 1: “Versatile” students learning through multiple and varied contexts, with strong support from Wikipedia, Blogs and YouTube (*n* = 108). The first cluster represents 19.1% of the total sample and is characterized by significantly high scores on most variables, the highest among all clusters, with no low scores. The learning ecologies of this group are rich, distributed, and conscious. Regarding the activities in which they participate, we find that this group has the highest score in formal face-to-face actions (courses and events), followed by remarkable scores in digital and informal actions, which can be used as complementary support for formal learning. Regarding resources, they use more formally guided online resources than formal print resources and UOC material, with little difference between the two. Regarding interactions, we found slightly higher scores with groups outside the academic field. Finally, their use of social media is very unequal: they make great use of platforms with static core and organizational content features (Wikipedia, Blogs and YouTube) and a more discreet use of social network platforms.

Learner profile 2: “Dependent” students with preference for academically guided learning resources (*n* = 107). The second cluster represents 19% of the total sample and is characterized by significantly high scores on variables in place with academically guided learning resources and a moderate score on the rest of the update resources, while especially low on those that have to do with professional contexts. Cluster 2 learning ecologies are strongly dependent on academic indicators. In relation to the learner activity component, we find a low participation in formal face-to-face actions and moderate participation in digital actions that are less formally guided. Regarding the resource’s component, we found a high score on formal resources on paper and material of the UOC, so it seems to rely basically on formal content offered from the academic field, which is supported by the other variable with high score, such as interacting with academic groups. In contrast, online resources and information source platforms occupy a neutral position and social network oriented platforms have the lowest negative score among the 5 clusters.

Learner profile 3: “Self-guided” students with preference for informal and digital learning resources and with support for interactive social network platforms (*n* = 113). The third cluster represents 20% of the sample and is characterized by high scores in survey variables that have to do with more informal and digital resources, actions and interaction and a moderate and low score on those more formal and analog resources. This group is in clear contrast from the previous cluster 2 profile. Cluster 3 learning ecologies are self-guided and happen through digital contexts. In relation to the learner activity component, we observed a high score on mostly digital and informal actions and a low score on formal face-to-face actions (courses and events). Regarding the resource’s component, the trend is repeated, showing a high score (the highest of all clusters) on less formally guided digital resources and a low score on formal resources on paper and UOC material. Regarding the learner interaction component, the cluster again displays a higher score in interactions with groups outside the academic realm than within it. Regarding the use of social media, unlike the previous cluster, we see a preference for social and interactive network platforms (Twitter, LinkedIn, Instagram, and Facebook) and discreet use of Wikipedia, YouTube, and blogs.

Learner profile 4: "Analog" students with preference for physical and formal resources, with some support for interactive social media platforms (*n* = 133). The fourth cluster represents 23.6% of the total sample, the highest percentage among all identified groups, and is characterized by moderate and high scores on variables that have to do with analog resources, physical resources, and interactions outside of an academic context and social network-type platforms and low scores on all other variables. Cluster 4 learning ecologies take place through face-to-face actions and slightly formal mixed resources, establishing connections on social networking platforms. Regarding the learner activity component, this cluster participates more in face-to-face and formal actions such as courses and events, than in digital and informal activities. As far as the resource component is concerned, we find that they use formal resources on paper and UOC material, making little use of online resources. Regarding the interaction component, learners interact more with groups outside the academic context than within it. However, the cluster makes considerable use of interactive social network platforms.

Learner profile 5: “Detached" students academically, with low use of resources for learning and occasional social media (*n* = 103). The fifth cluster represents 18.3% of the total sample and is characterized by moderate to low scores on the 8 questionnaire variables. In general, cluster 2 learning ecologies have a low degree of use of actions, resources, and connections for professional development, as this cluster demonstrated the lowest scores of all groups. To some extent, it reveals findings that are opposite to the cluster 1 profile. In relation to the activity component, we find that among the low values, this group participates more in formal face-to-face actions than in more informal digital actions. Regarding the resource component, it is the opposite, this learner profile makes slightly higher use of digital resources that are less formally guided. Also, in line with low engagement we find little interaction with teachers and students, and, to a lesser extent but also low, with professionals and friends. Finally, students in this cluster make sporadic use of social media.

## Discussion and conclusion

The survey study described in this article has explored the perceptions of communication students regarding the role of social media in their learning ecologies, identifying different profiles of students according to the way learners organize their learning and their use of social media platforms. This exploration has intended increasing knowledge about how social media can enhance professional updating depending on the profiles of the students in order to be able to carry out a more flexible, personalised, and collaborative training, a key element in today’s education (Sangrà, 2020; Romeu-Fontanillas et al., 2020). Results can provide in the future a proven way to profile-based distance educational services, especially those related to the training offered by educational institutions, in the stage of initial or continuing training of communication professionals, the efficiency of which could be monitored to further improve both educational services and educational quality.

In relation to the first research question, regarding what profiles of students make up the UOC communication studies according to the way in which they organize their learning and their use of social media, the PCA extracted 19 core component solutions spread over 4 large blocks, of which 8 were identified as appropriate for cluster analysis. 5 main profiles of students were obtained. Students in cluster 1, labelled as “versatile students”, learn through multiple contexts, which can be both digital and informal, both formal and informal; have more confidence in social media platforms of the information source type (Wikipedia, YouTube, Blogs) than those of the social network type or with a strong interactive component (Twitter, Instagram, Facebook, or LinkedIn). Students in cluster 2, labelled as “dependent students”, present a high use of learning resources and high degree of interaction within the academic field; on the contrary, there is low use of social or interactive networked platforms, face-to-face and formal actions, and interactions outside the academic field. Students in cluster 3, labelled as “self-guided students”, show preference for actions, resources and interactions of digital and informal contexts and interactive and networked social media; minority use of more formal resources guided by academia. Students in cluster 4, labelled as “analog students”, present slight preference for physical/analog and formal resources, actions, and relationships, as well as support for social networked platforms. Students in cluster 5, labelled as “detached students”, show little engagement with those resources of learning that come from academia and seem detached academically.

In relation to the second research question, on the role of social media in the post-graduate education of communication students at the UOC, these platforms play a notable role with a different degree of support during this stage of professional development, according to the different profiles of identified respondents. Most of the profiles include communication students that rely on social media platforms to support their learning. In the formative stage, virtual spaces are considered especially useful for locating content of interest or viewing useful video tutorials through blogs, Wikipedia, or YouTube (cluster of "versatile students") and consulting content in different digital formats, through platforms such as Instagram, Facebook, Twitter, or LinkedIn (cluster of "autonomous students" and cluster of "traditional students"). The results obtained in the current study that reveal the prominent position of numerous social media platforms by so many learner profiles can be explained by their blurred use across different personal, professional, and academic contexts (González-Sanmamed et al., 2018). Findings reveal a division between some platforms that we could designate as more sources of information (Wikipedia, blogs, YouTube) and more interactive and networked social media (Twitter, Facebook, LinkedIn). In general, social media has become an essential tool for post-graduates, particularly linked to the current communication context (Hamad et al., 2017; Wobalis, 2018). The greater or lesser use of these platforms is related to a predisposition to experience the possibilities of new tools or to position oneself digitally.

As contributions of the article we would like to highlight overcoming the lack of research on social media and professional updating that are focused on the communication sector (Salaverría, 2016), delving into its context, challenges and trends and placing its professionals at the center. We have also expanded the framework of learning ecologies (Jackson, 2019) to this professional sector, designing and applying quantitative research techniques and instruments that delve into the specific components that activate its professionals. On the other hand, the research has identified profiles of communication students according to the way they learn and their use of social media, which can inform educational institutions, about the different contexts in which their students learn, favoring a dialogue between university education and professional practice, which in fact is demanded by Casero-Ripollés et al. (2013). In a global way, research findings expand on claims made in the literature that reinforce the idea that social media offers learning opportunities in the context of higher education (Middleton & Beckingham, 2015; Rehm & Notten, 2016). In the formative stage of communication professionals we have found a different degree of use of social media platforms, with a division between platforms that we could designate as more sources of information (Wikipedia, blogs, YouTube) and more interactive social media of the type of social network (Twitter, Facebook, LinkedIn). This division can give clues to understand the contribution of social media in their learning ecologies. In this sense, the different profiles of communication students identified in this research can contribute to inform educational institutions about the role of social media in the professional development of communication professionals, which facilitates a dialogue between initial formal training and subsequent informal learning that takes place mostly through self-guided digital environments.

As for the limitations, the data collection of our investigation was prior to the outbreak of the pandemic, it was mainly carried out throughout 2019, a time when COVID-19 had not yet made an appearance. This has meant that the pandemic situation and its consequences have not been addressed in either the qualitative or the quantitative study. Obviously, this situation has also had a full impact on communication students. In this regard, the social media use of communication students was studied before the pandemic, at a distant educational environment, where the learning ecologies of distant learners can be significantly more complex than in a regular face-to-face university. For this reason, it would be interesting to see if the disruptive situation that COVID-19 has caused in the educational context, where citizens learn in hybrid and asynchronous environments (Sangrà, 2020) with a intense use of social media platforms (Wong et al., 2021), has impacted the way communication students learn, as well as how the role of social media impacted their learning ecologies in the training processes in higher education. Similarly, we have focused on the most popular platforms according to the reports of that year, and since then others such as Tik Tok or Twitch worthy of consideration have gained relevance. On the other hand, the intentional sampling in which we have delimited communication students, in the context of the Open University of Catalonia (UOC), entails a great deal of prudence in the generalization of results. However, the findings of this research can be of interest to higher education institutions to rethink the potential of social media in the training process, especially in the universities of the field of communication.

Overall, the quantitative study presented in this article is part of a broader mixed-method research in the form of a doctoral thesis, which is completed with a multiple case study of 10 relevant digital communicators.

## Data Availability

All data generated or analysed during this study will be included in the research thesis “Oportunidades de los medios sociales para la actualización de los profesionales de la comunicación: una investigación exploratoria desde las ecologías de aprendizaje”.
